# Rapamycin Regulates the Expression and Activity of Krüppel-Like Transcription Factor 2 in Human Umbilical Vein Endothelial Cells

**DOI:** 10.1371/journal.pone.0043315

**Published:** 2012-08-24

**Authors:** Qian Ma, Xiaomin Nie, Miao Yu, Zhijian Wang, Shiwei Yang, Dean Jia, Yujie Zhou

**Affiliations:** Department of Cardiology, Beijing Anzhen Hospital Affiliated to the Capital Medical University, Beijing, China; Center for Cancer Research, National Cancer Institute, United States of America

## Abstract

**Background:**

Although rapamycin has been reported to increase procoagulants and decrease anticoagulants in human umbilical vein endothelial cells (HUVECs), there is no significant difference in the incidence of stent thrombosis between patients with drug-eluting stents (DESs) and those with bare metal stents (BMSs). Krüppel-like transcription factor 2 (KLF2) has been identified as a key regulator of endothelial antithrombotic function. We hypothesized that rapamycin might induce the expression and activity of KLF2, thereby counteracting coronary endothelial dysfunction induced by DESs.

**Methods and Results:**

Expression of KLF2, tissue factor (TF) and endothelial NO synthase (eNOS) were assessed in HUVECs treated with rapamycin at concentrations of 2, 20, 200 and 2000 ng/ml for 24 and 48 hours without or with thrombin. Rapamycin strongly induced the expression and activity of KLF2 in high dose groups (p<0.01). Compared with control group, the expression of TF was increased by rapamycin, which inhibited the expression of eNOS after treating for 24 hours (p<0.01). Furthermore, small-interfering RNA–mediated knockdown of KLF2 strongly magnified the ability of rapamycin to induce TF and reduce eNOS accumulation in HUVECs.

**Conclusions:**

Rapamycin-dependent induction of KLF2 might partly counteract coronary endothelial dysfunction and thereby provided a novel molecular target to prevent stent thrombosis induced by DESs.

## Introduction

The treatment of coronary artery disease with percutaneous placement of drug-eluting stents (DESs) is associated with significant safety and a lower rate of restenosis compared to the placement of bare metal stents (BMSs) [1]–[2]. Rapamycin (sirolimus), which is widely used on DESs, produces many biological effects in the coronary circulation. For example, rapamycin released from DESs inhibits migration and proliferation of vascular smooth muscle cells (VSMCs) and endothelial cells, thereby preventing restenosis [3]–[4]. In vitro, proliferation of VSMCs and endothelial cells is suppressed by rapamycin via interference with several cell-cycle regulators involved in G1–S phase transition including p21cip1 [5], p27Kip1 [6], cyclin D isoforms, and the retinoblastoma protein (pRb) [3]. In vivo, DESs implantation leads to delay of reendothelialization leaving a highly prothrombotic surface exposed to the blood stream [7]–[8]. Furthermore, rapamycin inhibits homing, proliferation, and differentiation of endothelial progenitor cells (EPCs) by interacting with the mammalian target of rapamycin (mTOR), and delays proper endothelial regeneration [9]–[12]. Finally, rapamycin induces the expression of tissue factor (TF) [13]–[15] and plasminogen activator inhibitor 1 (PAI-1) [16]–[17]; on the other hand, rapamycin reduces the expression of endothelial NO synthase (eNOS) [18] and tissue-plasminogen activator (t-PA) [16]–[17]. As stated previously, it appears a potential increased risk for stent thrombosis with DESs as compared to BMSs.

Stent thrombosis remains a severe complication after stent implantation. Several factors are associated with an increased risk of stent thrombosis, including the procedure (stent malapposition and/or under expansion, number of implanted stents, stent length, persistent slow coronary blood flow), characteristics of the patient and lesion, stent itself (antiproliferative agents and/or polymers), and premature cessation of antiplatelet drugs [1]–[2]. Because rapamycin increases the expression of procoagulants and decreases the expression of anticoagulants, DESs has been thought to have a higher risk of stent thrombosis compared with BMSs. However, Laura Mauri applied a hierarchical classification of stent thrombosis set by the Academic Research Consortium (ARC) across randomized trials [19], which demonstrated that there is no significant difference in stent thrombosis incidence between DESs and BMSs.

Krüppel-like transcription factors (KLFs) is a subclass of the zinc-finger family of transcription factors characterized by the DNA binding domain containing the conserved sequence such as the “CACCC” or “GT-box” [20]. KLF2 has been identified as a key “molecular switch” that regulates important aspects of endothelial function and maintains an antithrombotic endothelial surface [20]. Overexpression of KLF2 strongly induces the expression of thrombomodulin (TM), eNOS and t-PA; on the other hand, KLF2 reduces the expression of PAI-1 and TF [20]–[21]. In contrast, siRNA mediated knockdown of KLF2 reduces antithrombotic gene expression while inducing the expression of procoagulants.

The expression of KLF2 in cytotoxic lymphocytes (CTLs) was negatively regulated by PI3K and mTOR. It has been demonstrated that mRNA expression of KLF2 was strikingly increased in CTLs treated with rapamycin [22]. Inhibition of mTOR induced KLF2 expression and subsequently increased the expression of KLF2 target genes such as CD62L and sphingosine 1 phosphate receptor 1(S1P1) [22].

Therefore, we hypothesized that rapamycin may counteract coronary endothelial dysfunction by inducing the expression and activity of KLF2, and KLF2 may play an important role in preventing stent thrombosis induced by rapamycin-eluting stents.

## Results

### 1. HUVECs Treated with Rapamycin Alone

#### 1.1 Rapamycin induces the expression of KLF2 in HUVECs

**Figure 1:**
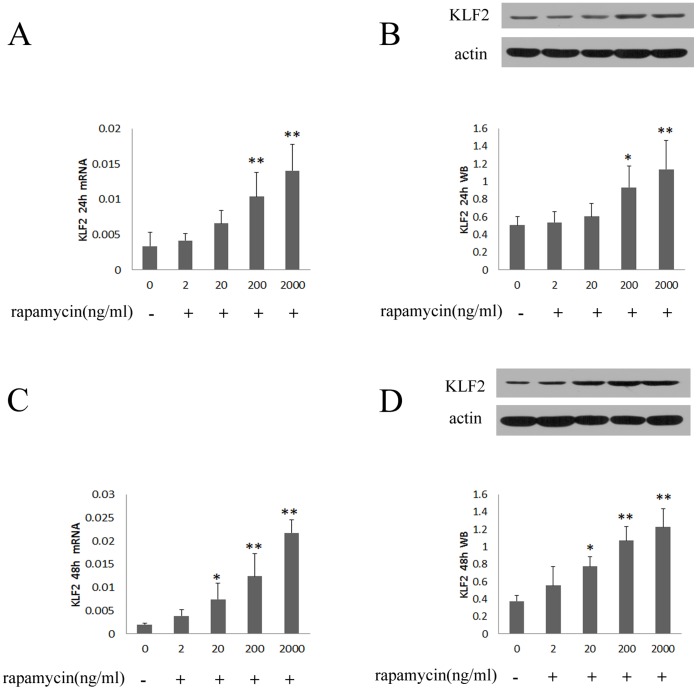
Rapamycin induced mRNA and protein expression of KLF2 in HUVECs. Rapamycin was added to HUVECs at concentrations of 2, 20, 200 and 2000 ng/ml for 24 and 48 hours. KLF2 was assessed by real-time PCR, western blot and immunofluorescence assays. Each bar represented the mean±SD (n = 3). Quantitative data for the PCR and western studies were shown graphically. A, rapamycin induced mRNA expression of KLF2 for 24 hours. B, rapamycin induced protein expression of KLF2 for 24 hours. C, rapamycin induced mRNA expression of KLF2 for 48 hours. D, rapamycin induced protein expression of KLF2 for 48 hours. Rapamycin enhanced the expression of KLF2 compared to control group (0 ng/ml) when the blood concentration of rapamycin was higher than 20 ng/ml (*p<0.05, **p<0.01 for rapamycin group vs control group).

Previous studies identified KLF2 as a key “molecular switch” that regulated the expression of TF, and eNOS [20]–[21]. In this study, we built on these initial observations and provided evidence that rapamycin induced the expression of KLF2 in HUVECs. Treating with rapamycin (200, 2000 ng/ml) for 24 hours enhanced mRNA and protein expression of KLF2 (Figure 1A–1B). The increase of mRNA expression of KLF2 was 3.1- and 4.2-fold for being treated with rapamycin at the concentration of 200 and 2000 ng/ml, respectively (Figure 1A). Protein expression of KLF2 was 1.8-and 2.2-fold after treating with rapamycin at the concentration of 200 and 2000 ng/ml (Figure 1B). There was also a significant increase in expression of KLF2 after the treatment of rapamycin for 48 hours (Figure 1C–1D).

#### 1.2 Rapamycin enhances the expression of TF in HUVECs

**Figure 2:**
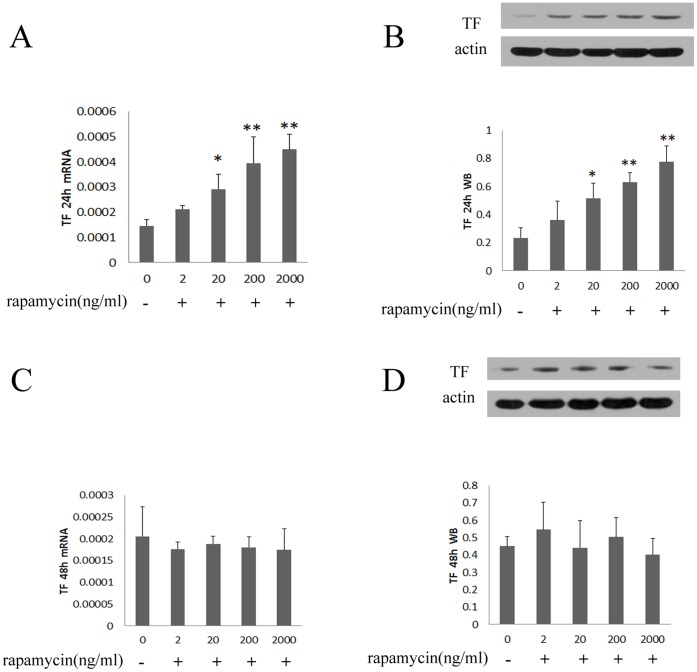
Rapamycin regulated mRNA and protein expression of TF in HUVECs. HUVECs were treated with rapamycin at final concentrations of 2, 20, 200 and 2000 ng/ml for 24 and 48 hours. TF was assessed by real-time PCR and western blot. Each bar represented the mean±SD (n = 3). Quantitative data for the PCR and western studies were shown graphically. A, rapamycin induced mRNA expression of TF for 24 hours. B, rapamycin induced protein expression of TF for 24 hours. C, rapamycin regulated mRNA expression of TF for 48 hours. D, rapamycin regulated protein expression of TF for 48 hours. Treating HUVECs with rapamycin for 24 hours resulted in a concentration-dependent increase of mRNA and protein expression of TF compared to control group (0 ng/ml). There was no significant change of mRNA and protein expression of TF after treating with rapamycin for 48 hours compared with control group (*p<0.05, **p<0.01 for rapamycin vs control group).

Treating HUVECs with rapamycin (20–2000 ng/ml) for 24 hours resulted in a concentration-dependent enhancement of TF compared with control group (Figure 2A–2B). However, there was no significant change of mRNA and protein expression of TF after treating with rapamycin for 48 hours (Figure 2C–2D).

#### 1.3 Rapamycin regulates the expression of eNOS in HUVECs

**Figure 3:**
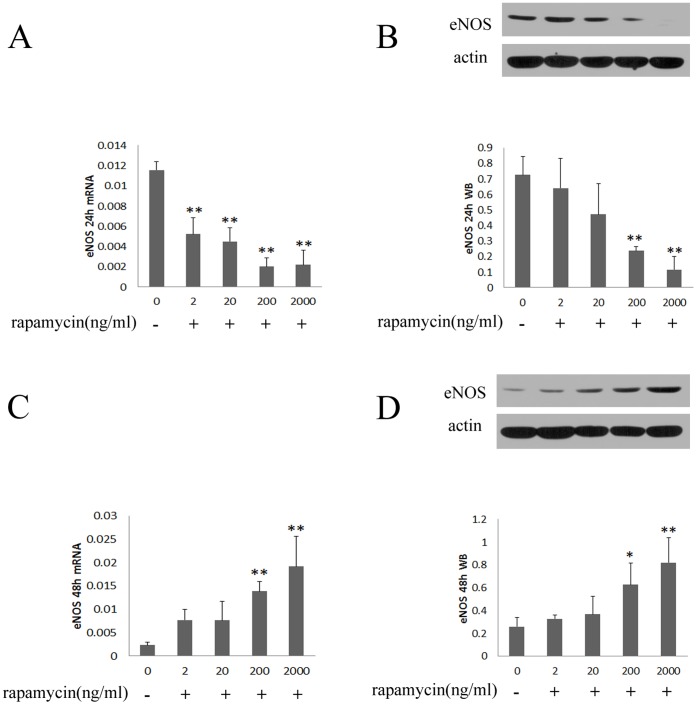
Rapamycin regulated mRNA and protein expression of eNOS in HUVECs. HUVECs were treated with rapamycin at concentrations of 2, 20, 200 and 2000 ng/ml for 24 and 48 hours. eNOS was assessed by real-time PCR and western blot. Each bar represented the mean±SD (n = 3). Quantitative data for the PCR and western studies were shown graphically. A, rapamycin reduced mRNA expression of eNOS for 24 hours. B, rapamycin reduced protein expression of eNOS for 24 hours. C, the mRNA expression of eNOS was induced after treating with rapamycin for 48 hours. D, the protein expression of eNOS was induced after treating with rapamycin for 48 hours. There was a significant decrease in mRNA and protein expression of eNOS after treating with rapamycin for 24 hours compared to control group (0 ng/ml). However, the expression of eNOS was enhanced by treating with rapamycin for 48 hours compared to control group. (*p<0.05, **p<0.01 for rapamycin vs control group).

As a key regulator of anticoagulants, eNOS was assessed in HUVECs treated with rapamycin. As shown in Figure 3A–3B, treating with rapamycin (2–2000 ng/ml) for 24 hours resulted in a concentration-dependent decrease of eNOS. However, the expression of eNOS was increased after being treated with rapamycin for 48 hours (Figure 3C–3D).

#### 1.4 Effect of KLF2 “Knockdown” on endothelial gene expression

**Figure 4:**
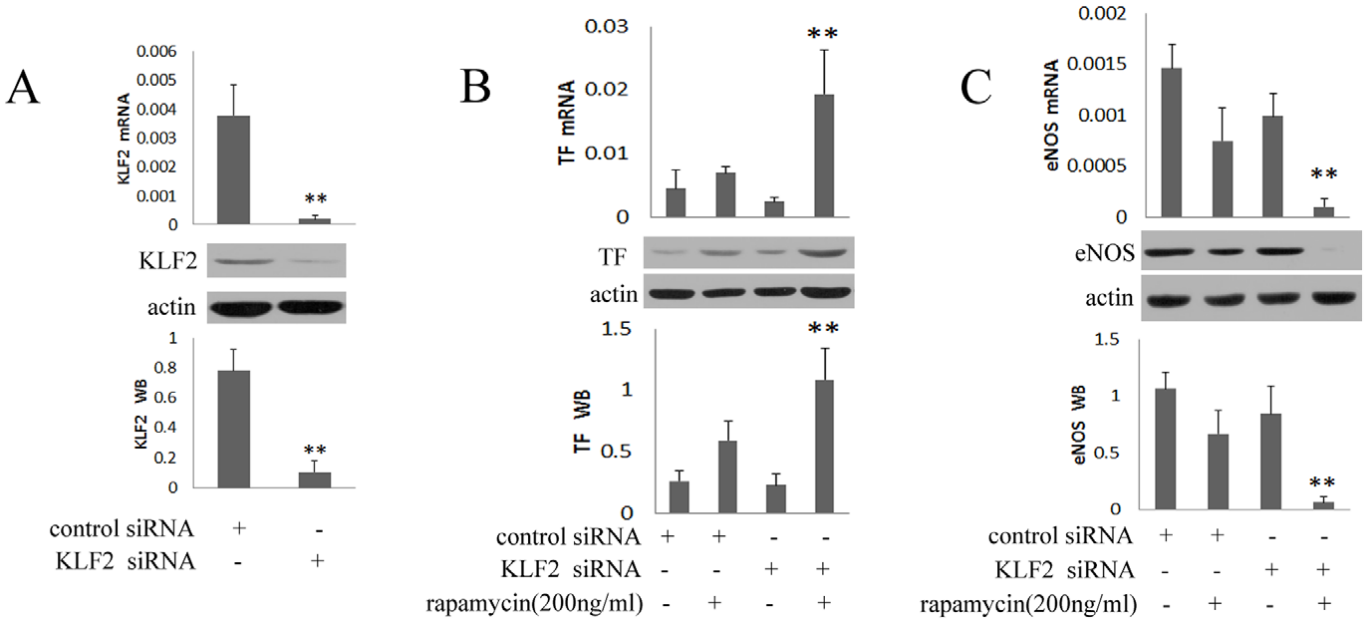
Effect of KLF2 “Knockdown” on Endothelial Gene Expression. After being transfected for 24 hours, HUVECs were treated with rapamycin at the concentration of 200 ng/ml for 24 hours. HUVECs were transfected with nonspecific siRNA (control siRNA) or specific siRNA (KLF2 siRNA) for 48 hours. The mRNA and protein levels of KLF2, TF and eNOS were assessed by real-time PCR and western blot. Each bar represented the mean±SD (n = 3). Quantitative data for the PCR and western studies were shown graphically. A, siRNA-mediated knockdown of KLF2 reduced the expression of KLF2. As shown in Figure A, a strong reduction in mRNA and protein expression of KLF2 was achieved with specific siRNA (**p<0.01 for KLF2 siRNA vs negative control siRNA). B, rapamycin induced mRNA and protein expression of TF after knockdown of KLF2. The 2.8-fold and 1.8-fold induction of mRNA and protein expression of TF by rapamycin were magnified after knockdown of KLF2 (**p<0.01 for KLF2 siRNA + rapamycin 200 ng/ml vs the negative control siRNA + rapamycin 200 ng/ml). C, rapamycin reduced mRNA and protein expression of eNOS after knockdown of KLF2. The 86% and 91% reduction of mRNA and protein expression of eNOS by rapamycin were magnified after knockdown of KLF2 (**p<0.01 for KLF2 siRNA + rapamycin 200 ng/ml vs control siRNA + rapamycin 200 ng/ml).

We performed siRNA-mediated KLF2 knockdown to assess the effect of KLF2 deficiency on endogenous TF and eNOS levels. As shown in Figure 4A, both mRNA and protein expression of KLF2 were significantly reduced by the transfection of specific KLF2 siRNA in HUVECs. The 2.8-fold and 1.8-fold induction of mRNA and protein expression of TF by rapamycin were magnified after knockdown of KLF2 (Figure 4B). In addition, the 86% and 91% reduction of mRNA and protein of eNOS by rapamycin were completely abrogated after knockdown of KLF2 (Figure 4C). These results indicate that siRNA–mediated knockdown of KLF2 strongly magnified the ability of rapamycin to increase TF and decrease eNOS accumulation in HUVECs.

### 2. HUVECs Treated with Thrombin and Rapamycin

#### 2.1 Rapamycin induces the expression of KLF2 in HUVECs with thrombin

**Figure 5:**
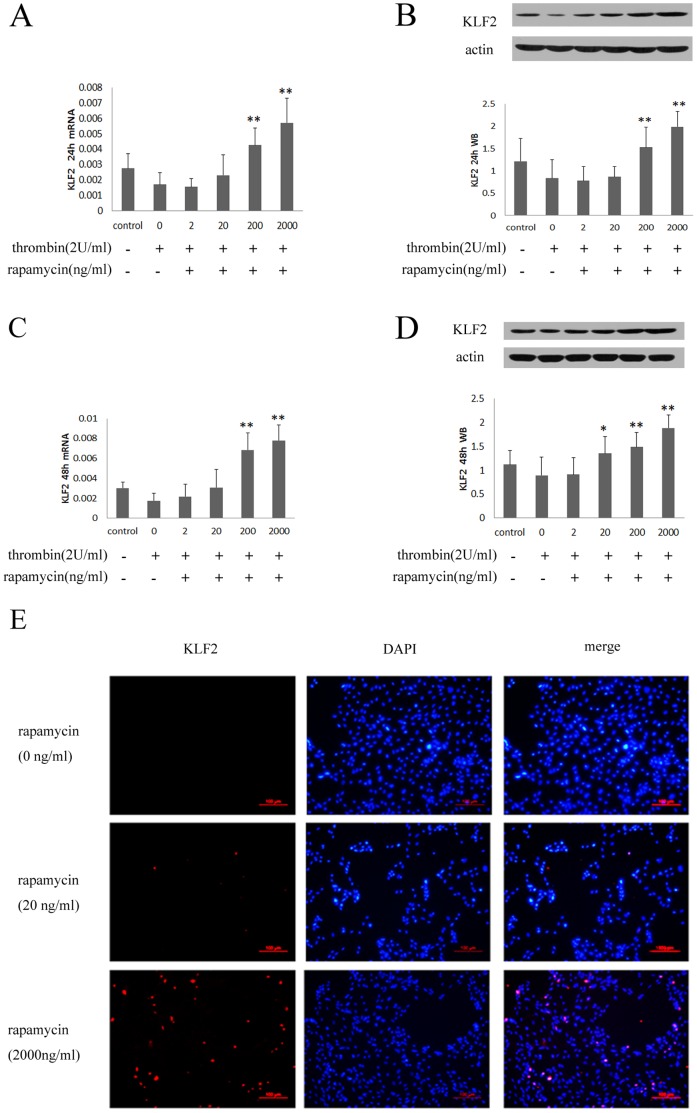
Rapamycin induced mRNA and protein expression of KLF2 in HUVECs with thrombin. After stimulation with thrombin for 4 hours, rapamycin was added to HUVECs at concentrations of 2, 20, 200 and 2000 ng/ml for 24 and 48 hours. KLF2 was assessed by real-time PCR, western blot and immunofluorescence assays. Each bar represented the mean±SD (n = 6). Quantitative data for the PCR and western studies were shown graphically. A, rapamycin induced mRNA expression of KLF2 for 24 hours. B, rapamycin induced protein expression of KLF2 for 24 hours. C, rapamycin induced mRNA expression of KLF2 for 48 hours. D, rapamycin induced protein expression of KLF2 for 48 hours. Rapamycin enhanced the expression of KLF2 compared to stimulation with thrombin alone when the blood concentration of rapamycin was higher than 20 ng/ml (*p<0.05, **p<0.01 for rapamycin + thrombin vs thrombin alone). E, immunofluorescence staining of KLF2 in the nuclei of HUVEC, controls or treated with rapamycin. KLF2 was shown in red (left column). DAPI was used to stain cell nuclei (center column). The merged image was shown in the right column.

Thrombin is generated by the cleavage of prothrombin at sites of vascular injury and it is induced after deployment of stents. In this study, we found that rapamycin induced the expression of KLF2 in HUVECs with thrombin. Treating with rapamycin (200, 2000 ng/ml) for 24 hours enhanced mRNA and protein expression of KLF2 (Figure 5A–5B). There was also a significant increase in expression of KLF2 after the treatment of rapamycin for 48 hours (Figure 5C–5D). We also detected the KLF2 migrating to the nucleus in an immunofluorescence analysis. We observed a concomitant increase of KLF2 in fluorescence with increasing concentrations of rapamycin (Figure 5E).

#### 2.2 Rapamycin regulates the activity of KLF2 in HUVECs with thrombin

**Figure 6:**
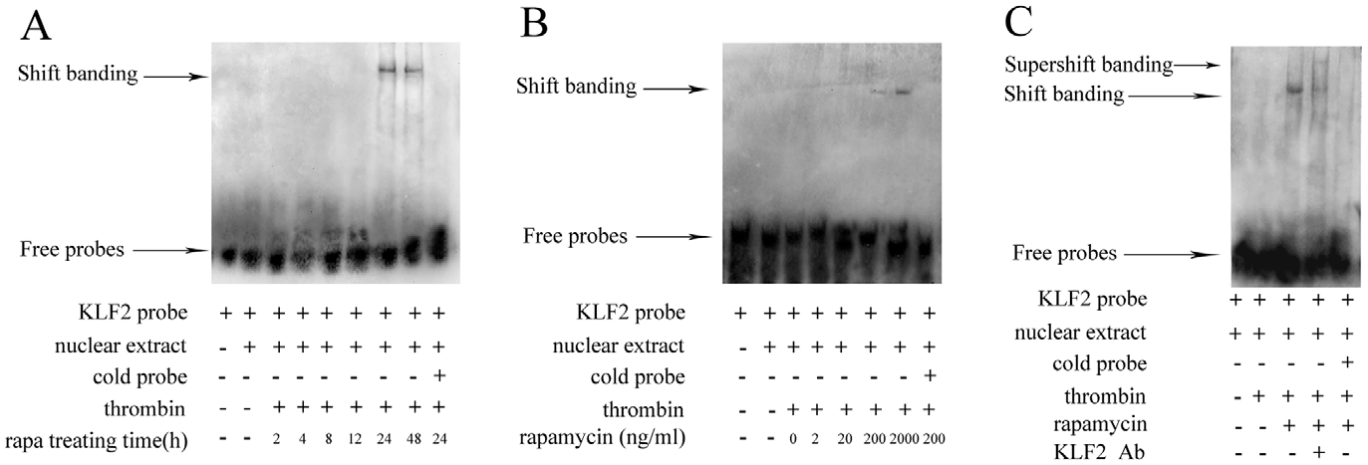
Rapamycin regulated the activity of KLF2 in HUVECs with thrombin. A, After stimulation with thrombin for 4 hours, rapamycin was added to HUVECs at the concentration of 200 ng/ml for 2, 4, 8, 12, 24 and 48 hours. The activity of KLF2 was assessed by EMSA. B, After stimulation with thrombin for 4 hours, HUVECs were treated with rapamycin at concentrations of 2, 20, 200 and 2000 ng/ml for 24 hours. C, After stimulation with thrombin for 4 hours, HUVECs were treated with rapamycin at concentrations of 2000 ng/ml for 24 hours or rapamycin + anti-KLF2 antibody. Compared to the control group (thrombin alone), KLF2 bound to specific DNA sequences after treating with rapamycin of 2000 ng/ml for 24 hours. Specificity was shown by supershift binding using anti-KLF2 antibody. A 100-fold molar excess of cold competitor oligomers reduced the detection of all complexes.

KLFs are transcription factors that bind DNA and regulate gene expression. Previous studies demonstrate that KLF2 can bind to sequences such as 5′-CACCC-3′, 5′-GTGGG-3′ (GT-box), or GC-rich sequences [20]. In order to determine whether the increase in the expression of KLF2 translated into an increase in function, we assessed the activity of KLF2 by EMSA. Nuclear extracts were incubated with biotin-labeled probes of KLF2. As shown in Figure 6A, biotin-labeled probes were able to strongly bind to the region of KLF2 promoter after treating with rapamycin at the concentration of 200 ng/ml for 24 and 48 hours. Shift bindings of KLF2-DNA complex were detected at the groups of 200 and 2000 ng/ml (Fig. 6B). The specificity of shift binding was verified by competition and supershift studies (Fig. 6C).

#### 2.3 Rapamycin enhances the expression of TF in HUVECs with thrombin

**Figure 7:**
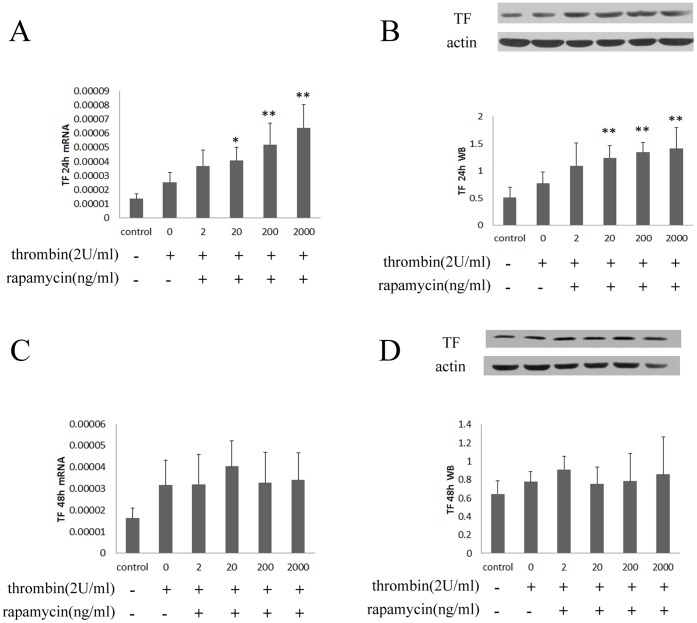
Rapamycin regulated mRNA and protein expression of TF in HUVECs with thrombin. After stimulation with thrombin for 4 hours, HUVECs were treated with rapamycin at final concentrations of 2, 20, 200 and 2000 ng/ml for 24 and 48 hours. TF was assessed by real-time PCR and western blot. Each bar represented the mean±SD (n = 6). Quantitative data for the PCR and western studies were shown graphically. A, rapamycin induced mRNA expression of TF for 24 hours. B, rapamycin induced protein expression of TF for 24 hours. C, rapamycin regulated mRNA expression of TF for 48 hours. D, rapamycin regulated protein expression of TF for 48 hours. Treating HUVECs with rapamycin for 24 hours resulted in a concentration-dependent increase of mRNA and protein expression of TF compared to stimulation with thrombin alone. There was no significant change of mRNA and protein expression of TF after treating with rapamycin for 48 hours compared with thrombin alone (*p<0.05, **p<0.01 for rapamycin+thrombin vs thrombin alone).

Treating HUVECs with rapamycin (20–2000 ng/ml) for 24 hours resulted in a concentration-dependent enhancement of TF with a maximal increase of 2.5-fold compared with control group (Figure 7A–7B). However, there was no significant change of mRNA and protein expression of TF after treating with rapamycin for 48 hours (Figure 7C–7D).

#### 2.4 Rapamycin reduces the expression of eNOS in HUVECs with thrombin

**Figure 8:**
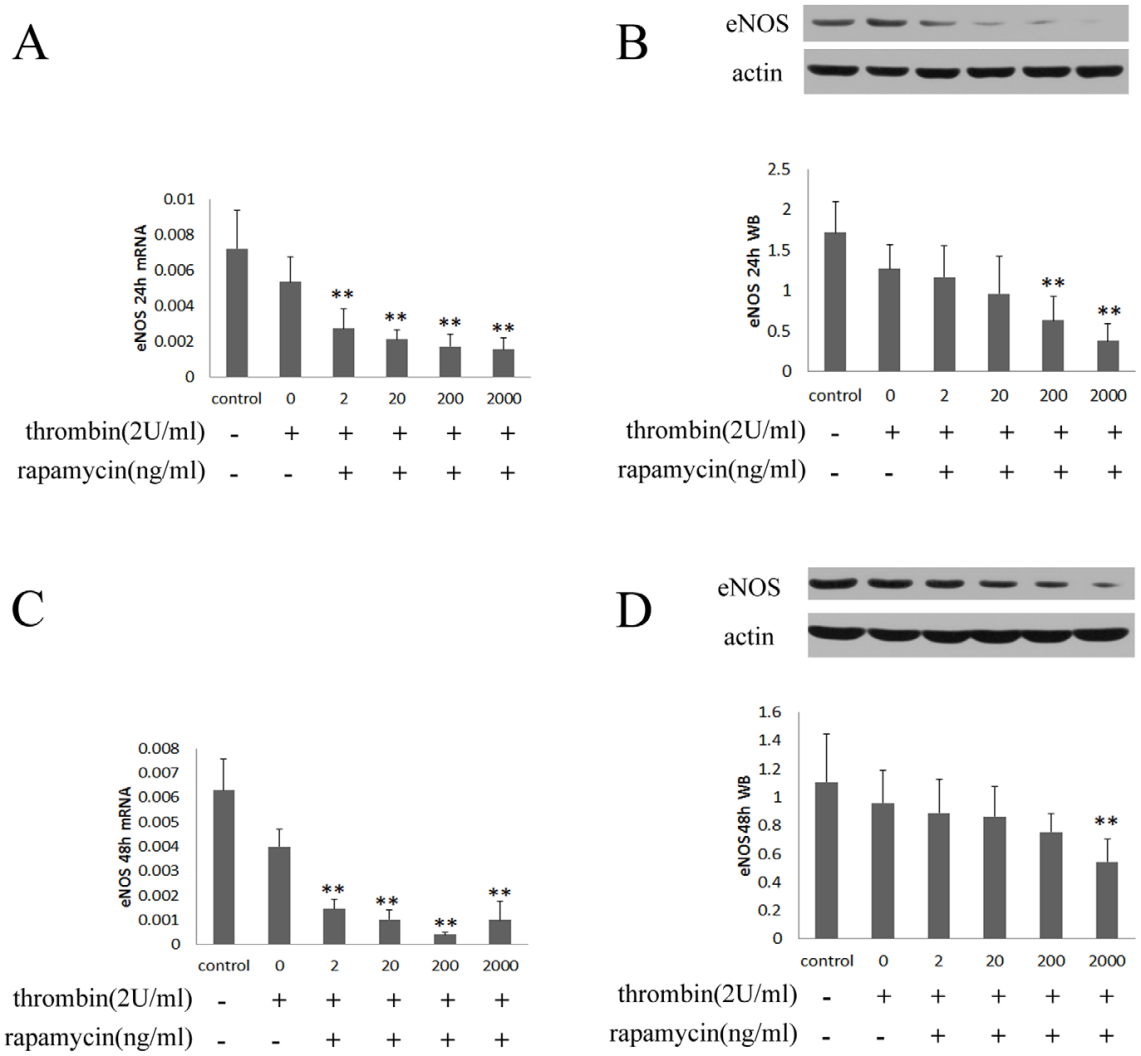
Rapamycin regulated mRNA and protein expression of eNOS in HUVECs with thrombin. After stimulation with thrombin for 4 hours, HUVECs were treated with rapamycin at concentrations of 2, 20, 200 and 2000 ng/ml for 24 and 48 hours. eNOS was assessed by real-time PCR and western blot. Each bar represented the mean±SD (n = 6). Quantitative data for the PCR and western studies were shown graphically. A, rapamycin reduced mRNA expression of eNOS for 24 hours. B, rapamycin reduced protein expression of eNOS for 24 hours. C, rapamycin reduced mRNA expression of eNOS for 48 hours. D, rapamycin reduced protein expression of eNOS for 48 hours. There was a significant decrease in mRNA and protein expression of eNOS after treating with rapamycin for 24 and 48 hours compared to stimulation with thrombin alone. (**p<0.01 for rapamycin + thrombin vs thrombin alone).

As shown in Figure 8A–8B, treating with rapamycin (2–2000 ng/ml) for 24 hours resulted in a concentration-dependent decrease of eNOS. The mRNA and protein expression of eNOS were also decrease after being treated with rapamycin for 48 hours (Figure 8C–8D).

#### 2.5 Effect of KLF2 “Knockdown” on endothelial gene expression with thrombin

**Figure 9:**
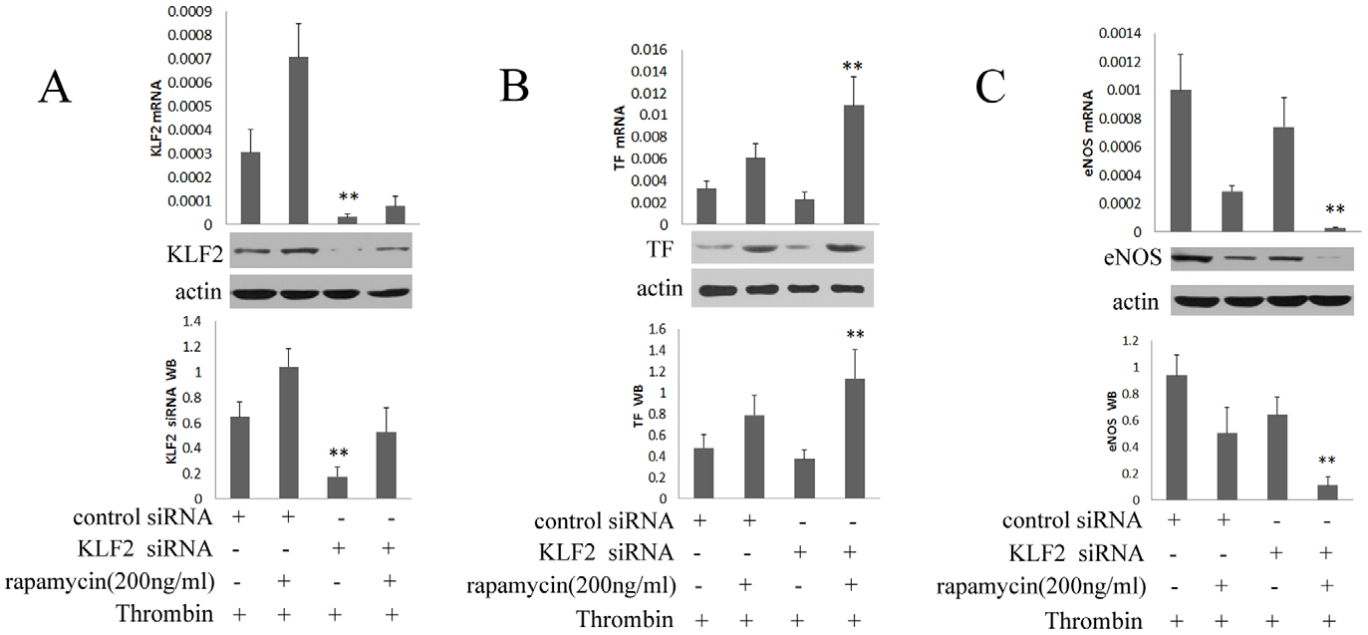
Effect of KLF2 “Knockdown” on Endothelial Gene Expression with thrombin. After being transfected for 20 hours, HUVECs were stimulated with thrombin for 4 hours, and treated with rapamycin at the concentration of 200 ng/ml for 24 hours. HUVECs were transfected with nonspecific siRNA (control siRNA) or specific siRNA (KLF2 siRNA) for 48 hours. The mRNA and protein levels of KLF2, TF and eNOS were assessed by real-time PCR and western blot. Each bar represented the mean±SD (n = 6). Quantitative data for the PCR and western studies were shown graphically. A, siRNA-mediated knockdown of KLF2 reduced the expression of KLF2. As shown in Figure A, a strong reduction in mRNA and protein expression of KLF2 was achieved with specific siRNA (**p<0.01 for KLF2 siRNA vs negative control siRNA). B, rapamycin induced mRNA and protein expression of TF after knockdown of KLF2. The 1.8-fold and 1.4-fold induction of mRNA and protein expression of TF by rapamycin were magnified after knockdown of KLF2 (**p<0.01 for KLF2 siRNA + rapamycin 200 ng/ml vs the negative control siRNA + rapamycin 200 ng/ml). C, rapamycin reduced mRNA and protein expression of eNOS after knockdown of KLF2. The 90% and 78% reduction of mRNA and protein expression of eNOS by rapamycin were magnified after knockdown of KLF2 (*p<0.05, **p<0.01 for KLF2 siRNA + rapamycin 200 ng/ml vs control siRNA + rapamycin 200 ng/ml).

As shown in Figure 9A, both mRNA and protein expression of KLF2 were significantly reduced by the transfection of specific KLF2 siRNA in HUVECs. The basal level of TF and eNOS were reduced by 30%. The 1.8-fold and 1.4-fold induction of mRNA and protein expression of TF by rapamycin were magnified after knockdown of KLF2 (Figure 9B). Furthermore, the 90% and 78% reduction of mRNA and protein of eNOS by rapamycin were completely abrogated after knockdown of KLF2 (Figure 9C). These results indicated that siRNA–mediated knockdown of KLF2 magnified the ability of rapamycin to increase TF and decrease eNOS accumulation in HUVECs with thrombin.

## Discussion

In this study, we provided initial evidence that rapamycin induced the expression and activity of KLF2 in HUVECs and explained the role of KLF2 in preventing stent thrombosis induced by rapamycin-eluting stents.

Serious concerns about the security of DESs, especially stent thrombosis, developed after the study of BASKET-LATE [23]–[29]. However, these studies used nonuniform definitions of stent thrombosis and had limited power to detect low-frequency events [29]–[34]. Thus, Laura Mauri implemented a new standardized, hierarchical definition of stent thrombosis for uniform evaluation of events in a pooled analysis of eight randomized trials [19]. During the four years follow-up, the incidence of stent thrombosis did not differ significantly between patients with DESs and those with BMSs.

Although rapamycin-eluting stents were designed to elute 80% of the drug by 30 days, rapamycin easily penetrated cell walls owing to its lipophilic property, leading to retention in arterial tissue [35]–[37]. Even though rapamycin has been reported to increase TF and PAI-1, and decrease eNOS and t-PA, rapamycin did not raise more incidence of stent thrombosis. KLF2 can regulate some key factors involved in maintaining an antithrombotic endothelial surface [21].

Thrombin is generated at sites of vascular injury and is induced at the statement of atherosclerosis and stents implantation. Zhiyong Lin et al found that thrombin binding its receptor PAR-1 could induce several signaling pathways that converge on a number of transcriptional mediators [38]. In endothelial cells, thrombin can alter the expression of multiple factors that collectively confer a prothrombotic phenotype. For example, thrombin can induce the expression of TF and PAI-1. Furthermore, thrombin can inhibit the expression of factors such as eNOS and endothelin-1. Finally, treatment with thrombin decreases the expression of KLF2 in HUVECs.

To examine the role of KLF2 in rapamycin-depended thrombosis, the expression of KLF2, TF and eNOS were assessed in HUVECs treated with rapamycin alone at different concentrations for 24 and 48 hours. We found that the expression of KLF2 was strongly induced by rapamycin. And HUVECs treated with rapamycin for 24 hours enhanced the expression of TF and reduced the expression of eNOS in a concentration-dependent manner. By virtue of high expression of KLF2, there was no significant change of the expression of TF after treating with rapamycin for 48 hours. Furthermore, the expression of eNOS was increased after treating for 48 hours because of the enhancement of KLF2. Data showed that rapamycin induced the expression and activity of KLF2 when the concentrations of rapamycin were high, and may counteract coronary endothelial dysfunction induced by rapamycin.

Secondly, we performed the dose-dependent experiments with rapamycin and thrombin in order to approach more precisely to the statement of atherosclerosis and stents implantation. Similarly, treating with rapamycin (200–2000 ng/ml) for 24 and 48 hours enhanced mRNA and protein expression of KLF2 (p<0.05 to <0.01). Although KLF2 directly induced eNOS, both rapamycin and thrombin reduced the expression of eNOS in endothelial cells. Consequently, the mRNA and protein expression of eNOS was still lower after being treated with rapamycin for 48 hours. Data showed that rapamycin induced the expression and activity of KLF2 at the statement of atherosclerosis and stents implantation, and may partly prevent stent thrombosis induced by DESs.

On a subcellular level, rapamycin binded to the FK-binding protein 12 and subsequently inhibits mTOR [14]–[15], a downstream target of the phosphatidylinositol-3 kinase (PI3K) pathway [39]–[41]. Therefore, rapamycin inhibited the PI3K/Akt/mTOR pathway subsequently increasing the expression and activity of TF in response to TNF-α, histamine, thrombin, and VEGF [42]–[44]. Furthermore, other studies showed that high level of PI3K signals downregulated the expression and promoter activation of KLF2 [45]–[47]. Therefore, KLF2 may be a downstream target of the PI3K/Akt/mTOR pathway, regulating the expression of endothelial procoagulants and anticoagulants, and rapamycin may induce the expression and activity of KLF2 by inhibiting the PI3K pathway.

The concentrations of rapamycin occurring in vivo were identical with those used in our study. The maximal systemic concentration of rapamycin after deployment of two stents was reported to be nearly 1 ng/ml [48]. However, the local concentration around the stents was significantly higher (80–200 ng/ml). The lipophilic property of rapamycin led to its accumulation in the arterial wall [36], [48], [49], so we used 2000 ng/ml as the maximal concentration. Because an in vitro study of HUVECs couldn’t represent the complex biology of the in vivo situation, we intend to further investigate the clinical relevance of the present findings.

Recently, several studies demonstrated that statins (e.g. mevastatin, simvastatin, and lovastatin) could induce KLF2 in a concentration-dependent manner [50]–[52]. Consequently, statins might be considered for patients who had high risk of stent thrombosis in order to induce KLF2 and prevent stent thrombosis.

The author(s) disclosed receipt of the following financial support for the research, authorship, and/or publication of this article: supported by the National Nature Science Foundation of China (#30971238).

## Materials and Methods

### Endothelial Cell Culture and Rapamycin-mediated Infection

HUVECs were obtained from ScienCell an0064 cultured as described previously [17]. HUVECs were cultured under 37°C and 5% CO2 in the endothelial cell medium (ECM, ScienCell) according to the manufacturer’s instruction. The ECM was consisted of 500 ml of basal medium, 25 ml of fetal bovine serum, 5 ml of endothelial cell growth supplement and 5 ml of penicillin/streptomycin solution. Rapamycin (LC laboratories) was added to the culture medium at final concentrations of 2, 20, 200 and 2000 ng/ml, respectively. HUVECs treated with rapamycin at multiple concentrations were incubated for 24 and 48 hours. For all groups, n = 3. Then, HUVECs were stimulated with thrombin (Sigma, 2 U/ml) for 4 hours so that it was close to the statement of atherosclerosis and stents implantation. HUVECs treated with rapamycin at multiple concentrations were incubated for 24 and 48 hours again. For all groups, n = 6.

### Immunofluorescence Assays

The cover slips were washed with phosphate buffered saline (PBS) for three times. Then, cells were fixed in 4% paraformaldehyde and incubated with 10 mM ammonium chloride for 10 min. Cells were then rinsed with PBS for three times, blocked with 2.5% normal goat serum in Tween-20 in PBS (PBST), and then incubated with KLF2 antibody (Santa Cruz, sc-28675, 1∶50) at 4°C for 12 hours. Red Alexa Fluor 594-conjugated goat anti-rabbit IgG (Fab) (1∶720) was incubated for 1 hour at 37°C.

### Real-Time Quantitative RT-PCR

HUVECs were lysed, RNA isolated, and real-time quantitative RT-PCR performed as described previously [17]. We prepared purified and intact total RNA from HUVECs using the SV total RNA isolation system according to the manufacturer’s recommendations. The total RNA was reverse-transcribed to cDNA using the GoScript™ reverse transcriptase. The real-time PCR was performed using BioRad iQ™5 (Hercules) and SYBR® Premix Ex Taq™ purchased from Takara Belmont. Fold changes in mRNA expression were calculated using the 2^–ΔΔCt^ method. The sequences of the specific primers were referred to GenBank and designed as follows: KLF2: sense, 5′-TGAGAAGCCCTACCACTGCAAC-3′, antisense, 5′-GCACAGATGGCACTGGAATG-3′;TF: sense,5′-CTACTGTTTCAGTGTTCAAGCAGTGA-3′,antisense,5′-CAGTGCAATATAGCATTTGCAGTAGC-3′;eNOS:sense,5′-TTCCGCTACCAGCCAGACC-3′,antisense,5′-CACTCGCTTCGCCATCACC-3′;GAPDH:sense,5′-TGGTCTCCTCTGACTTCAAC-3′,antisense,5′-GTGAGGGTCTCTCTCTTCCT-3′.

### Western Blot

HUVECs were lysed in 50 µl of radioimmunoprecipitation assay (RIPA) lysis buffer with inhibitors of protease and phosphatase. Each lane was loaded with 20–30 µg of protein. Protein was separated by SDS-PAGE (8%) and transferred to a PVDF membrane. Primary antibody of KLF2 (Abcam, ab28526) was used for 12 hours at 1∶500 dilution (4°C); antibodies of TF (Abcam, ab88387) and eNOS (Abcam, ab76198) were used at 1∶1000 dilution. Blots were incubated with horseradish peroxidase-labeled secondary antibodies for 1 hour. Immunoreactive bands were detected with enhanced chemiluminescence.

### Nuclear Extract and Electrophoretic Mobility Shift Assay (EMSA)

10 µg of nuclear extract was used per 20 µl of binding reaction. Probe of KLF2 was designed as follows: forward, 5′-ATCCACAGAGACCAGCCCATTTCTTAG-3′; reverse, 5′-TAGGTGTCTCTGGTCGGGTAAAGAATC-3′. Biotin-labeled probe of KLF2 (300 fmol) was added to the mixture of reaction for 20 minutes at 20°C. Supershift was performed with polyclonal antibody of KLF2 (Santa Cruz, sc-28675, 10 µg/binding reaction). Protein-DNA complex was separated from free DNA probe by electrophoresis in a nondenaturing polyacrylamide gel. After transferring and crosslinking, we detected biotin-labeled DNA by Chemiluminescence.

### Transient Transfection Assays

HUVECs were plated in 6-well plates for 24 hours before transfection. The sequences of siRNA-KLF2 were GCACCGACGACGACCUCAAUU, UCAACAGCGUGCUGGACUUUU, and UGCUGGAGGCCAAGCCAAAUU. 30 nM of RNAi (Santa Cruz, sc-35818) duplex was transiently transfected with Lipofectamine™ RNAiMAX transfection reagent (Invitrogen). After being transfected for 20 hours, HUVECs were stimulated with thrombin for 4 hours, and treated with rapamycin for 24 hours at the concentration of 200 ng/ml. Cells were harvested after transfection for 48 hours (n = 6).

### Statistical Analysis

All data were expressed as mean±SD. We used one-way ANOVA to analyze the differences of variables. A two-tailed value of P<0.05 was considered as statistically significant difference. **P<0.01, *P<0.05 between the indicated groups. All statistical analyses were performed with SPSS 18.0.
